# Supplementary material for: Correlated-photon imaging at 10 volumetric images per second
Source: Sci Rep. 2023 Aug 7;13:12813. doi: 10.1038/s41598-023-39416-8 (PMC10406932; doi:10.1038/s41598-023-39416-8)
Supplement: Supplementary file 1 — Supplementary Information. [file 41598_2023_39416_MOESM1_ESM.pdf]

SUPPLEMENTARY INFORMATION
Correlated-photon imaging at 10 volumetric images per second

Gianlorenzo Massaro[1,2,4], Paul Mos[3,4], Sergii Vasiukov[2,4], Francesco Di Lena[2,5], Francesco Scattarella[1,2,5], Francesco V. Pepe[1,2,*], Arin Ulku[3], Davide Giannella[1,2], Edoardo Charbon[3], Claudio Bruschini[3,6], and Milena D'Angelo[1,2,6]

[1]Università degli studi di Bari, Dipartimento Interuniversitario di Fisica, Bari, I-70126, Italy
[2]Istituto Nazionale di Fisica Nucleare, Sezione di Bari, Bari, I-70125, Bari, Italy
[3]Ecole polytechnique fédérale de Lausanne (EPFL), Neuchâtel, 2002, Switzerland
[4]Equal contribution
[5]Equal contribution
[6]Equal contribution
[*]francesco.pepe@ba.infn.it

ABSTRACT

The document contains information that corroborates the findings presented in the main article text.

S1 Plenoptic properties of the intensity correlation function

As demonstrated in Ref.[1], plenoptic information is encoded in the correlation function of Eq. (1) in the main text, representing the correlation between the intensity fluctuations reaching two points, of which one is placed on the detector D_a, and the other on the detector D_b. The correlation function reads, up to irrelevant factors that do not depend on either $\boldsymbol{\rho}_a$ or $\boldsymbol{\rho}_b$,

$$\Gamma(\boldsymbol{\rho}_a,\boldsymbol{\rho}_b) = \left| \iint A(\boldsymbol{\rho}_o) A^*(\boldsymbol{\rho}'_o) \Psi(\boldsymbol{\rho}_o,\boldsymbol{\rho}'_o,\boldsymbol{\rho}_a,\boldsymbol{\rho}_b) d^2\boldsymbol{\rho}_o d^2\boldsymbol{\rho}'_o \right|^2, \tag{S1}$$

where A is the aperture function of the object. The function Ψ can be considered as a "second-order point-spread function", which determines the correspondence between object points and detector points. By referring to the parameters in Fig. 3 of the main text, and assuming the source emits chaotic light with an average intensity profile $S(\boldsymbol{\rho}_s)$ and negligible transverse coherence, we have

$$\Psi(\boldsymbol{\rho}_o,\boldsymbol{\rho}'_o,\boldsymbol{\rho}_a,\boldsymbol{\rho}_b) = p_a(\boldsymbol{\rho}_o,\boldsymbol{\rho}_a) p_b(\boldsymbol{\rho}'_o,\boldsymbol{\rho}_b) \int S(\boldsymbol{\rho}_s) e^{\frac{ik}{2z\sigma}\left[\boldsymbol{\rho}_o^2-(\boldsymbol{\rho}'_o)^2-2(\boldsymbol{\rho}_o-\boldsymbol{\rho}_o')\cdot\boldsymbol{\rho}_s\right]} d^2\boldsymbol{\rho}_s, \tag{S2}$$

with the functions p_j, $j=a,b$, describing field propagation from the object to the detector. Calling P the pupil function of the lens, we obtain

$$p_j(\boldsymbol{\rho}_o,\boldsymbol{\rho}_j) = \int P(\boldsymbol{\rho}_{\ell j}) e^{ik_z\phi_j(\boldsymbol{\rho}_o,\boldsymbol{\rho}_{\ell j},\boldsymbol{\rho}_j)} d^2\boldsymbol{\rho}_{\ell j} \tag{S3}$$

with

$$\phi_j(\boldsymbol{\rho}_o,\boldsymbol{\rho}_{\ell j},\boldsymbol{\rho}_j) = \left(\frac{1}{z-z_j+z_o} - \frac{1}{z_o}\right)\frac{\boldsymbol{\rho}_{\ell j}^2}{2} - \left(\frac{\boldsymbol{\rho}_o}{z-z_j+z_o} - \frac{\boldsymbol{\rho}_j}{M z_o}\right)\cdot\boldsymbol{\rho}_{\ell j} \quad . \tag{S4}$$

Notice that the differences in phases ϕ_j with respect to the original results described in Ref.[1] are due to the fact that the optical paths are split upstream of the lens, and not downstream.

The plenoptic properties of the correlation function are easily deduced, for example, from the fact that, by applying a stationary-phase approximation to the integrals which defines it, one obtains

$$\Gamma(\boldsymbol{\rho}_a,\boldsymbol{\rho}_b) \sim \left| A\left[\frac{(z_b-z)\boldsymbol{\rho}_a-(z_a-z)\boldsymbol{\rho}_b}{M\Delta z}\right]\right|^4. \tag{S5}$$

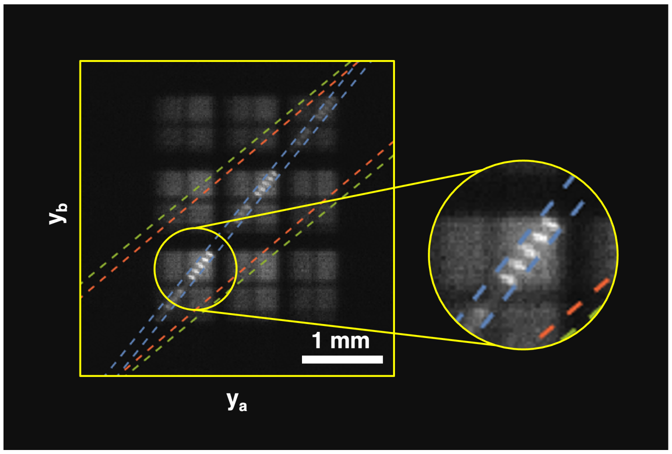

Figure S1. Two-dimensional correlation function obtained after integration along the x direction of data acquired by the two sensors in the experiment corresponding to case B (as reported in Fig. 2 of the main text). Dashed green and blue lines identify the region (defined by the lens) where non-zero correlations can be found. The dotted blue lines identify the correlated region defined by the light source profile. The overall correlation region is given by the intersection of the three regions.

Therefore, $\Gamma(\boldsymbol{\rho}_a, \boldsymbol{\rho}_b)$ represents a collection of images of the object, whose shifts and scaling depend on the axial position of the latter. The refocusing process, consisting in properly realigning the images contained in the correlation function, is discussed in the next section.

S2 Refocusing and correlation aperture

In a ray-optics approximation, the correlation function that is measured reads[1–3]:

$$\begin{aligned}\Gamma(\boldsymbol{\rho}_a, \boldsymbol{\rho}_b) = & \left| A\left[\frac{(z_b - z)\boldsymbol{\rho}_a - (z_a - z)\boldsymbol{\rho}_b}{M\Delta z}\right]\right|^4 \\ & \times \left| P\left[\frac{(z_o + \Delta z)\boldsymbol{\rho}_a - z_o\boldsymbol{\rho}_b}{M\Delta z}\right]\right|^2 \left| P\left[\frac{z_o\boldsymbol{\rho}_a - (z_o - \Delta z)\boldsymbol{\rho}_b}{M\Delta z}\right]\right|^2 \\ & \times \left| S\left[\frac{(z_b - z_\sigma)\boldsymbol{\rho}_a - (z_a - z_\sigma)\boldsymbol{\rho}_b}{M\Delta z}\right]\right|^2 \quad . \end{aligned} \tag{S6}$$

This equation shows that Γ contains information regarding the object aperture A, and that also the lens aperture P and source intensity profile S have effects on the correlation function. Each aperture defines a region inside the correlation space $(\boldsymbol{\rho}_a, \boldsymbol{\rho}_b)$, as shown in Fig. S1: the green and red lines indicate the regions where non-zero correlations can be expected, as defined by the lens; the blue lines, instead, indicate the effects of limited aperture of the source, *i.e.* the width of the Gaussian profile illuminating the GGD. In fact, correlation can only be measured if the light passes through all the apertures, and this means that the correlation region is the intersection of the three correlation regions defined by P and S. By considering only the correlation region, in data analysis, one eliminates all the spurious signal outside that area, which can only be due to noise. Moreover, to simplify the numerical operations, one can consider only the smallest of the regions defined by P and S rather than their intersection (in our case, the area defined by the source). In order to determine the dominating aperture, an effective radius in the correlation space should be evaluated for all the apertures. For the lens of aperture P we consider a circular 2D pupil function with radius equal to the effective radius of the lens: $r_\ell = \mathrm{NA}_o z_o$. If we call *correlation aperture* (CA) the radius of the region defined by the lens, we obtain

$$\mathrm{CA}_{\mathrm{lens},a} = \mathrm{NA}_o \frac{|M\Delta z|}{\sqrt{(1 + \Delta z/z_o)^2 + 1}} \qquad \mathrm{CA}_{\mathrm{lens},b} = \mathrm{NA}_o \frac{|M\Delta z|}{\sqrt{(1 - \Delta z/z_o)^2 + 1}}. \tag{S7}$$

The same quantity can be defined and calculated for the source:

$$\mathrm{CA}_{\mathrm{source}} = \frac{r_\sigma}{z_\sigma} \frac{|M\Delta z|}{\sqrt{(z_b/z_\sigma - 1)^2 + (z_a/z_\sigma - 1)^2}} \quad , \tag{S8}$$

where $r_\sigma = c\sigma = 1.44$ mm is the radius of the source aperture, that depends on the standard deviation $\sigma = 1.02$ mm of its Gaussian profile. The factor c is used as an optimization parameter to maximize the SNR. To show that the source is the limiting aperture in our experimental conditions, we report the three radii:

$$\mathrm{CA}_{\mathrm{lens},a} = 0.63\,\mathrm{mm}, \quad \mathrm{CA}_{\mathrm{lens},b} = 0.53\,\mathrm{mm}, \quad \mathrm{CA}_{\mathrm{source}} = 0.07\,\mathrm{mm}. \tag{S9}$$

The refocusing $\alpha(z)$ is a linear operator that transforms the coordinates on the detector planes $\boldsymbol{\rho}_a$ and $\boldsymbol{\rho}_b$ into two new ones: $\boldsymbol{\rho}_r$ related to the refocusing plane, and $\boldsymbol{\rho}_s$ related to another generic plane in z_s. It reads:

$$\alpha(z) : \begin{bmatrix} \boldsymbol{\rho}_a \\ \boldsymbol{\rho}_b \end{bmatrix} \mapsto \begin{bmatrix} \boldsymbol{\rho}_r \\ \boldsymbol{\rho}_s \end{bmatrix} = \frac{1}{M\Delta z} \begin{bmatrix} z_b - z & z - z_a \\ z_b - z_s & z_s - z_a \end{bmatrix} \begin{bmatrix} \boldsymbol{\rho}_a \\ \boldsymbol{\rho}_b \end{bmatrix} \quad . \tag{S10}$$

By applying the transformation to the measured correlation function in the position z of the target, one obtains

$$\Gamma_r(\boldsymbol{\rho}_r, \boldsymbol{\rho}_s, z) = \Gamma\left(\alpha^{-1}(z) \begin{bmatrix} \boldsymbol{\rho}_r \\ \boldsymbol{\rho}_s \end{bmatrix}\right) \sim |A(\boldsymbol{\rho}_r)|^4 |S(\boldsymbol{\rho}_s)|^2 \quad . \tag{S11}$$

The last approximate equality stems from the much smaller radius of the source with respect to the lens, so that the latter can be considered irrelevant. We should point out, however, that the algorithm of Eq.(S10) is the most convenient choice only when the source is the limiting aperture and should be modified accordingly if the lens becomes the dominating aperture. The final integration

$$\Sigma(\boldsymbol{\rho}_r, z) = \int \Gamma_r(\boldsymbol{\rho}_r, \boldsymbol{\rho}_s, z) d^2\boldsymbol{\rho}_s \sim |A(\boldsymbol{\rho}_r)|^4 \tag{S12}$$

can be done by limiting the integration domain to an area defined by the source radius.

By comparing Eqs. (S6) and (S10), we see that the coefficients on which the object aperture depends correspond to the first row of the refocusing matrix, while the second row contains the coefficients that appear in the source profile. The first line gives us an insight into what the refocusing algorithm actually does, that is, rescaling the detector coordinates so that, in the transformed plane, the object depends only on the set of two-dimensional coordinates $\boldsymbol{\rho}_r$. It is also clear that the second line, defining the variable $\boldsymbol{\rho}_s$ that is integrated in Eq. (S12), plays no relevant role for the object reconstruction, and can be chosen arbitrarily. However, some choices are more convenient than other when defining the integration variable. For example, since the extension of the correlation function is defined by the size of the apertures, a clever choice is to define $\boldsymbol{\rho}_s$ as the transverse coordinate of one of the limiting apertures. By doing so, one can integrate only where non-zero correlations are expected, and limit the integration of Eq. (S12) to a much smaller area than what has been measured, so as to speed up calculations quite dramatically. Given the values of the correlation apertures in Eq. (S9), it made sense to us to choose the integration variable as the one defined by the source. If that were not the case, the second line would have been modified with the coefficients of the aperture defining the major limitation on the correlation area.

S3 Data analysis workflow

Data analysis has been performed on MATLAB and consisted of two main parts, that are data reading and correlation, and refocusing. In the first part, the binary frames are read from the disk and the correlation function is evaluated by averaging over all frames. Computation of the correlation function is fast and efficient, so that the speed of the process was mostly defined by the data read rate of the disk; reading $\sim 10^4$ frames (512×256) and computing the correlation function took about 200 s. After the correlation function is available in the workstation memory as a 4D array, the operation of refocusing took about 14 s per axial coordinate z.

S4 Study of the SNR dependence on the number of frames

We report in Fig. S2 the full analysis of the dependence of the SNR on the number of frames N_t. The analysis has been performed in the experimental conditions corresponding to case "C" of Fig. 2 (main text). The experimental points indicates that the SNR tends to saturate as the number of frames increases. This behavior deviates from the $\sqrt{N_t}$ scaling, expected if the collected frames were statistically independent, since our source can only provide a finite number of samples. We thus fit the experimental points of the SNR with the function

$$\mathrm{SNR}(N_t) = \frac{1}{\sqrt{a + \frac{b}{N_t}}}, \tag{S13}$$

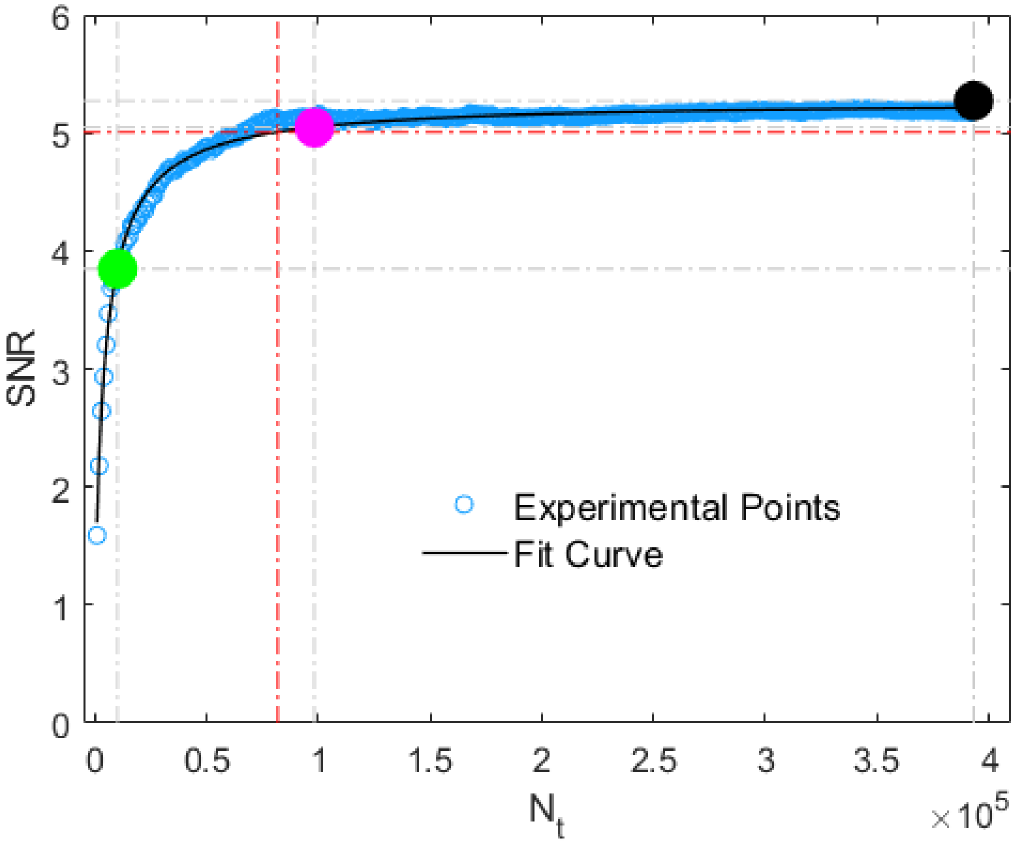

Figure S2. Behavior of the SNR in the image refocused by CPI (referred to case C shown in Fig. 3 of the main text), as a function of the number of collected frames N_t. Blue circles represent the SNR evaluated on the experimental refocused images, while the solid black line corresponds to the fitting curve $(a+b/N_t)^{-1/2}$. The points discussed in the text, and corresponding to $N_t = 4.0\times10^5$, $N_t = 9.8\times10^4$, and $N_t = 9.8\times10^3$, are highlighted in black, magenta, and green, respectively. The red dotted lines correspond to $N_t = 8\times10^4$, where we estimated a decrease of the SNR by 5% of its maximum.

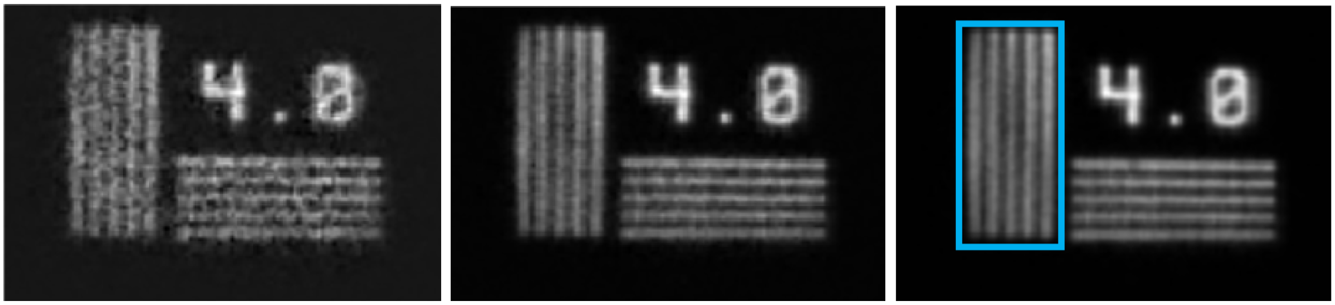

(a) $N_t = 9.8 \times 10^3$, SNR = 3.9 (b) $N_t = 9.8 \times 10^4$, SNR = 5.1 (c) $N_t = 4.0 \times 10^5$, SNR = 5.3

Figure S3. Comparison of the refocused images acquired at 10 (a) and 1 (b) volumetric images per second. Panel (c) shows the refocused image using the whole acquired dataset. The cyan rectangle indicates the area where the SNR has been evaluated on the three images.

which accounts for both the $\sqrt{N_t}$ behavior at a small number of frames and the saturation at a high number of frames. The best fit provides $a = 3.60 \times 10^{-2}$ and $b = 3.18 \times 10^2$. The plot in Fig. S2 indicates that variations in the number of acquired frames around $N_t = 4.0 \times 10^5$ does not yield an appreciable improvement in image quality. Actually, a reduction of N_t by a factor close to 4 ($N_t = 9.8 \times 10^4$) with respect to the whole dataset, which enables to collect each CPI image in one second ($T_{\mathrm{CPI}} = 1\,\mathrm{s}$), produces a decrease in the SNR by less than 5%. Moreover, a further reduction in the number of frames to $N_t = 9.8 \times 10^3$, which enables to collect 10 CPI in one second ($T_{\mathrm{CPI}} = 0.1\,\mathrm{s}$), Fig. S3 shows a comparison between these three cases, with the corresponding estimated values of the SNR. The SNR in the region of interest enclosed in the cyan rectangle highlighted in Fig. S3, has been estimated as

$$\mathrm{SNR} = \frac{\overline{\Sigma}_{\mathrm{in}}}{\Delta_{\mathrm{in}}\Sigma}, \tag{S14}$$

where $\overline{\Sigma}_{\mathrm{in}}$ represents the average value of the signal, in the image, in correspondence of the transmissive parts of the considered five-slit group; the denominator $\Delta_{\mathrm{in}}\Sigma$ represents the standard deviation of the values contributing to the numerator. Such a definition relies on the realistic assumption that the statistical distributions of the signal in the refocused image, in correspondence of the transmissive parts, are identical.

References

1. Di Lena, F. *et al.* Correlation plenoptic imaging between arbitrary planes. *Opt. Express* **28**, 35857–35868 (2020).
2. Pepe, F. V., Scarcelli, G., Garuccio, A. & D'Angelo, M. Plenoptic imaging with second-order correlations of light. *Quantum Meas. Quantum Metrol.* **3**, 20–26 (2016).
3. Massaro, G., Di Lena, F., D'Angelo, M. & Pepe, F. V. Effect of finite-sized optical components and pixels on light-field imaging through correlated light. *Sensors* **22**, 2778 (2022).
